# Triglyceride-glucose–body mass index as an integrated marker for ultrasound-defined diastolic dysfunction: variable importance via machine learning and SHAP analysis in a Chinese community cohort

**DOI:** 10.3389/fcvm.2026.1952042

**Published:** 2026-10-08

**Authors:** Pengcheng Li, Jing Bai, Jirui Cai, Jingyi Zhang, Zhiwei Huang, Jin Wang, Bing He, Qiaotao Xie, Haoran Wang

**Affiliations:** 1 Department of Cardiology, the First Affiliated Hospital of Zhengzhou University, Zhengzhou University, Zhengzhou, China; 2 Cardiovascular Institute of Luohe, Luohe Central Hospital, Luohe Medical College, Luohe, China

**Keywords:** diastolic dysfunction, echocardiography, HFpEF, insulin resistance, machine learning, obesity, triglyceride-glucose–body mass index

## Abstract

**Introduction:**

The triglyceride-glucose–body mass index, which integrates the triglyceride-glucose index with body mass index, has emerged as a promising marker of metabolic risk for heart failure with preserved ejection fraction; however, its association with subclinical diastolic dysfunction remains underexplored.

**Methods:**

This cross-sectional study enrolled 1,467 participants from a Chinese community-based population who underwent echocardiographic evaluation and metabolic profiling. The triglyceride-glucose–body mass index was calculated as the natural logarithm of triglycerides multiplied by fasting blood glucose divided by 2, then multiplied by body mass index. Ultrasound-defined diastolic dysfunction was diagnosed according to the 2025 American Society of Echocardiography criteria. Multivariable logistic regression, restricted cubic spline analysis, and machine learning were employed to evaluate the association.

**Results:**

The prevalence of ultrasound-defined diastolic dysfunction was 33.6%. The optimal Youden-derived triglyceride-glucose–body mass index cutoff was 220.000, categorizing participants into low (n=543) and high (n=924) groups. In the fully adjusted model, each 1-standard deviation increase was not significantly associated with ultrasound-defined diastolic dysfunction in continuous analysis (odds ratio=1.35; 95% confidence interval: 0.42–4.40; P=0.616); however, the binary comparison showed a significant association (odds ratio=1.60; 95% confidence interval: 1.11–2.31; P=0.013). Restricted cubic spline analysis revealed a significant overall association ($P_{\mathrm{overall}}<0.001$) without significant nonlinearity ($P_{\mathrm{nonlinear}}=0.194$).

**Discussion:**

In this cohort, the triglyceride-glucose–body mass index demonstrated a significant independent association with ultrasound-defined diastolic dysfunction in the binary analysis, supporting its potential as a screening index for subclinical diastolic dysfunction, although external validation in independent populations is needed.

## 1 Introduction

Heart failure with preserved ejection fraction (HFpEF) accounts for roughly one-half of all heart failure cases worldwide and exhibits a prognosis as severe as heart failure with reduced ejection fraction (1). Over recent decades, the rising prevalence of HFpEF has been driven primarily by an aging population and the growing burden of cardiometabolic conditions such as obesity, diabetes, and hypertension. Managing HFpEF remains a significant clinical challenge due to high morbidity, frequent hospital admissions, and historically limited treatment options that have largely targeted symptom relief rather than disease progression.

Diastolic dysfunction, defined by impaired left ventricular relaxation and increased filling pressures, constitutes the core pathophysiological abnormality in HFpEF and often emerges years before overt symptoms develop (2). This condition spans a spectrum from grade I (impaired relaxation) through grade III (restrictive filling), reflecting progressive myocardial stiffening and structural remodeling. Early-stage diastolic dysfunction is frequently asymptomatic, rendering early detection clinically important yet difficult. Population-based investigations have established that subclinical diastolic dysfunction, even without heart failure symptoms, independently predicts cardiovascular events, mortality, and progression to clinically manifest HFpEF (3).

Metabolic comorbidities—obesity, diabetes, and dyslipidemia—are central to HFpEF pathogenesis through interconnected mechanisms (4, 5). The conceptualization of HFpEF has expanded from a purely cardiac disorder to a systemic inflammatory condition wherein cardiometabolic risk factors provoke coronary microvascular endothelial inflammation, initiating myocardial structural changes and dysfunction. Among these factors, dyslipidemia has received growing recognition as a modifiable contributor to cardiac impairment. Elevated triglycerides (TG), reduced high-density lipoprotein cholesterol (HDL-C), and elevated remnant cholesterol have each been linked to subclinical diastolic dysfunction in community-based studies (6–8). Nevertheless, individual lipid measures capture only partial aspects of lipid metabolism and may inadequately represent the comprehensive metabolic stress imposed by coexisting lipid derangements and central adiposity.

The TG/HDL-C ratio has gained acceptance as a straightforward and robust indicator of insulin resistance and atherogenic dyslipidemia (9–12). Epidemiologic evidence indicates that this ratio more strongly correlates with cardiometabolic risk than isolated lipid components, reflecting the combined impact of triglyceride-rich lipoproteins and diminished HDL-mediated protection. Meanwhile, overall obesity, commonly assessed via body mass index (BMI), predicts cardiovascular events and metabolic syndrome beyond body mass index (BMI) alone, capturing visceral adiposity and its associated inflammatory and metabolic disturbances (13).

The Triglyceride-Glucose–Body Mass Index (TyG-BMI), computed as ln(TG×FBG/2)×BMI , integrates the TyG index (a validated marker of insulin resistance) with body mass index (a measure of overall adiposity) into a single metric that concurrently reflects atherogenic dyslipidemia and central adiposity. Originally proposed as a superior discriminator for diabetes and cardiovascular risk in East Asian populations (14–16), TyG-BMI has demonstrated enhanced predictive value for cardiovascular events relative to individual cardiometabolic markers. By jointly quantifying insulin resistance (via the TyG index) and the metabolic consequences of overall obesity (via BMI), TyG-BMI may better encapsulate the overall cardiometabolic stress that drives subclinical cardiac dysfunction through systemic inflammation, endothelial impairment, and myocardial lipotoxicity.

Despite the biological rationale linking cardiometabolic dysfunction to diastolic impairment, no prior investigation has examined the association between TyG-BMI and echocardiographically defined diastolic dysfunction. Moreover, the application of machine learning methods to corroborate the independent predictive utility of TyG-BMI in diastolic function evaluation remains unexplored. Machine learning approaches provide advantages over conventional statistics by managing high-dimensional data, capturing non-linear relationships, and delivering interpretable feature importance through techniques such as SHapley Additive exPlanations (SHAP).

In this study, we aimed to: (1) evaluate the association between TyG-BMI and ultrasound-defined diastolic dysfunction (USDD) using multivariable logistic regression; (2) characterize the dose-response relationship between TyG-BMI and USDD using restricted cubic spline analysis; (3) assess the consistency of this association across clinically relevant subgroups; and (4) validate the independent predictive value of TyG-BMI using multiple machine learning algorithms with SHAP-based interpretation.

## 2 Methods

### 2.1 Study population

Participants were drawn from the Longitudinal Investigation of Osteoarthritis and Cardiovascular Health Status cohort, a community-based study conducted in Luohe City, Henan Province, China (17). The the Longitudinal Investigation of Osteoarthritis and Cardiovascular Health Status cohort recruited middle-aged and older adults (35–75 years) from local communities, targeting a demographic with elevated prevalence of both cardiovascular conditions and osteoarthritis. Detailed descriptions of the cohort design, recruitment strategy, and baseline phenotyping protocols—including echocardiography, vascular ultrasonography, and comprehensive metabolic profiling—have been published previously (17).

Eligibility for the present analysis required: (1) continuous residence in the Luohe catchment area for ≥5 years; (2) capacity and consent to undergo cardiac and carotid ultrasound examinations; and (3) willingness to participate in longitudinal follow-up of at least 5 years’ duration.

Individuals were excluded if they met any of the following conditions: (1) acute myocardial infarction complicated by cardiogenic shock, end-stage heart failure, or hemodynamic compromise from any etiology; (2) active malignancy or severe traumatic injury (e.g., major burns, motor vehicle collisions) that threatened survival or substantially curtailed life expectancy; (3) significant psychiatric illness, altered consciousness, or cognitive impairment precluding valid questionnaire completion; (4) hemodynamically significant valvular disease, atrial fibrillation, or rhythm disturbances that would confound echocardiographic diastolic assessment; (5) echocardiographic window inadequate for reliable diastolic function grading.

Written informed consent was secured from all participants following a structured face-to-face discussion with trained study personnel. The protocol received approval from the Institutional Review Board of Luohe Central Hospital (No. 2023010) and was prospectively registered with the Chinese Clinical Trial Registry (ChiCTR2300071492). This report adheres to the Strengthening the Reporting of Observational Studies in Epidemiology (STROBE) cross-sectional checklist (18).

Enrollment spanned November 2023 through November 2024. Of 1,762 individuals who satisfied eligibility criteria and provided consent, the final analytic sample comprised 1,467 participants after excluding those with missing LVEF or TyG-BMI data. All remaining analyses used complete-case methods within each regression model.

Sample size estimation assumed a USDD prevalence of 34%, a relative odds of 1.3 comparing high vs. low TyG-BMI strata, two-tailedα=0.05, and 80% power, producing a minimum requirement of approximately 1,100 participants.

### 2.2 Variable definitions

#### 2.2.1 Triglyceride-glucose–body mass index (TyG-BMI)

TyG-BMI was calculated using the formula: TyG-BMI=ln(TG × FBG/2) × BMI, where TG (triglycerides) and FBG (fasting blood glucose) are expressed in mmol/L, and BMI is expressed in kg/m². This formula integrates the TyG index (a validated marker of insulin resistance) with body mass index (a measure of overall adiposity) into a single metric that captures both insulin resistance and overall adiposity. Higher TyG-BMI values indicate greater cardiometabolic risk, with proposed thresholds distinguishing low and high cardiometabolic risk profiles.

### 2.3 Clinical data collection

A standardized clinical workup was performed for each participant, comprising structured interview, physical examination, and fasting venipuncture. Demographic characteristics (age, sex, educational attainment, occupation), tobacco use (current: ≥1 cigarette/day in the preceding month; former; never), alcohol intake (regular: ≥1 occasion/week over the past year), and habitual physical activity were documented via interviewer-administered questionnaire.

Prevalent medical conditions were ascertained by self-report with corroboration from available health records. Operational definitions were as follows: hypertension — systolic blood pressure (SBP) ≥140 mmHg, diastolic blood pressure (DBP) ≥90 mmHg, or current antihypertensive pharmacotherapy; diabetes mellitus — fasting glucose ≥7.0 mmol/L, 2-h post-challenge glucose ≥11.1 mmol/L, HbA1c ≥6.5%, or glucose-lowering medication use; coronary heart disease (CHD) — prior myocardial infarction, ≥50% epicardial coronary stenosis on angiography, or coronary revascularization; stroke — ischemic or hemorrhagic subtype confirmed by CT/MRI; heart failure — diagnosed per prevailing clinical practice guidelines; atrial fibrillation — documented on 12-lead electrocardiography.

Anthropometry followed a rigorous, duplicate-measurement protocol. Stature was recorded to 0.1 cm with a wall-fixed stadiometer (barefoot, Frankfurt plane alignment). Body mass was obtained to 0.1 kg on a calibrated digital platform (light indoor attire). Waist girth was measured at the mid-point between the inferior costal margin and the superior iliac crest at end-expiration using a flexible, non-stretch tape. Body mass index (BMI) was derived as weight/height${}^{2}$ (kg/m${}^{2}$). The mean of two determinations was used.

Seated blood pressure was taken after ≥5 min of quiet rest with a mercury sphygmomanometer and an appropriately sized cuff. Of three successive readings obtained at 1-min intervals, the average of the latter two was retained for analysis.

Venous blood was drawn after an overnight fast (≥8 h). Serum total cholesterol (TC), triglycerides (TG), high-density lipoprotein cholesterol (HDL-C), and low-density lipoprotein cholesterol (LDL-C) were quantified by automated enzymatic assays. Fasting plasma glucose was determined by the glucose oxidase method.

### 2.4 Echocardiographic assessment

Transthoracic echocardiography was conducted by credentialed cardiac sonographers on Philips EPIQ 7C or GE Vivid E95 platforms with phased-array transducers, following a standardized imaging protocol aligned with ASE/EACVI chamber quantification recommendations (19). Sonographers completed uniform training and periodic quality audits. All acquisitions were obtained with the participant in the left lateral decubitus position.

Left ventricular ejection fraction (LVEF) was computed from M-mode tracings using the Teichholz formula: $\mathrm{LVEF}\;=\;[1\;-\;(\mathrm{LVIDs}/\mathrm{LVIDd}{)}^{3}]\;\times \;100\text{\%}$, supplemented by biplane Simpson’s method from apical four- and two-chamber views when endocardial definition permitted.

Left atrial (LA) volume was derived by the area-length technique from orthogonal apical views at end-systole (the frame immediately preceding mitral valve opening). LA volume index (LAVI) was calculated as LA volume normalized to body surface area, employing a threshold of >34 mL/m${}^{2}$ for LA enlargement in both sexes.

Diastolic function was graded according to the 2025 ASE algorithm (20). Tissue Doppler imaging sampled mitral annular motion at the septal and lateral insertion sites; early diastolic velocities (e′) were averaged across both annuli. Age-stratified thresholds for impaired relaxation were: average e′<9 cm/s (20–39 years), <7 cm/s (40–65 years), and <6.5 cm/s (>65 years). Transmitral inflow was recorded by pulsed-wave Doppler at the leaflet tips to measure early (E) and late (A) diastolic velocities, from which the E/A ratio and E/ e′ ratio (using average e′) were derived. An E/ e′ ratio >14 was taken to indicate elevated LV filling pressure.

Three ancillary markers contributed to the diagnostic algorithm: (1) E/A ≤0.8 or ≥2; (2) average E/ e′ >14; and (3) LAVI >34 mL/m${}^{2}$. USDD was diagnosed when either (a) age-specific e${}^{\prime }$ was reduced plus ≥1 ancillary marker was abnormal, or (b) e′ was preserved yet ≥2 ancillary markers were abnormal. All remaining patterns were classified as normal diastolic function (20, 21).

Offline readings were performed by experienced interpreters blinded to all clinical and biochemical data. Intra- and inter-observer reproducibility, evaluated in a random subset of 50 examinations, yielded intraclass correlation coefficients >0.85 for all primary diastolic metrics.

### 2.5 Statistical analysis

Analyses were performed in R version 4.4.2. Two-sided *P* < 0.05 was regarded as statistically significant throughout. Given the skewed distributions of most continuous variables, these were summarized as median [interquartile range (IQR)]; categorical data were expressed as count (percentage). Between-group comparisons of baseline characteristics employed the Wilcoxon rank-sum test for continuous measures and the $\chi^{2}$ test for categorical measures.

An optimal TyG-BMI cut-point was identified from the receiver operating characteristic (ROC) curve via Youden’s index (sensitivity + specificity − 1), and participants were subsequently stratified into low- and high TyG-BMI categories using this threshold.

Candidate predictors of USDD were first screened by univariable logistic regression; variables attaining *P* < 0.10 advanced to multivariable modeling. Three sequential logistic models were fitted to quantify the independent contribution of TyG-BMI:

- Model 1 (Crude): TyG-BMI alone
- Model 2 (Demographic-adjusted): TyG-BMI + age + sex
- Model 3 (Fully adjusted): TyG-BMI + age + sex + TG + HDL-C + current smoking + alcohol use+ hypertension + diabetes + CHD + stroke

Effect estimates are reported as odds ratios (OR) with 95% confidence intervals (CI). Model discrimination was summarized by the area under the ROC curve (AUC), with 95% CIs derived from 1,000 bootstrap resamples.

The dose-response relationship was characterized by restricted cubic spline (RCS) regression with knots positioned at the 10th, 50th, and 90th percentiles of the TyG-BMI distribution. Likelihood-ratio tests assessed the overall significance of the spline term ($P_{\mathrm{overall}}$) and departures from linearity ($P_{\mathrm{nonlinear}}$).

To evaluate consistency across population segments, prespecified subgroup analyses were conducted stratified by age (<60 vs. ≥60 years), sex, hypertension, diabetes, BMI (<24 vs. ≥24 kg/m${}^{2}$), and smoking status. Interaction was tested by introducing a cross-product term into the fully adjusted model; P_interaction_ < 0.05 was taken as evidence of effect modification.

Sensitivity analyses excluded participants with prevalent cardiovascular disease (CHD, stroke, heart failure, or atrial fibrillation) to assess whether the TyG-BMI–USDD association persisted in an ostensibly disease-free subset.

### 2.6 Machine learning analysis

To corroborate the regression findings with an independent computational framework, nine supervised classifiers were benchmarked under 5-fold stratified cross-validation: Logistic Regression (LR, serving as the conventional reference), Extra Trees (ET), LightGBM, eXtreme Gradient Boosting (XGBoost), CatBoost, Gradient Boosting Machine (GBM), K-Nearest Neighbors (KNN), Adaptive Boosting (AdaBoost), and Decision Tree (DT). Each algorithm underwent hyperparameter optimization via grid search with internal cross-validation to balance predictive accuracy and generalizability.

Features were preselected through univariable screening followed by variance inflation factor (VIF) diagnostics; predictors with VIF < 10 were retained. For parsimonious feature selection, least absolute shrinkage and selection operator (LASSO) regression was implemented with 5-fold cross-validation to select the optimal regularization parameter; features with non-zero coefficients at the selected penalty (best C = 0.041) were retained, and the remaining candidate predictors were excluded. Discriminative performance was quantified by the area under the ROC curve (AUC) averaged over five repeated cross-validation folds. Model interpretability was pursued through SHapley Additive exPlanations (SHAP). Global feature importance rankings and individual-level SHAP dependence curves were generated from the top-performing model. The SHAP=0 transition point for TyG-BMI was identified as the value at which the predicted probability of USDD crossed the population mean—providing a data-driven risk threshold complementary to the Youden-derived cut-point.

## 3 Results

### 3.1 Study population and baseline characteristics

A total of 1,467 participants drawn from the Longitudinal Investigation of Osteoarthritis and Cardiovascular Health Status community cohort were included in the final analytic sample, after excluding individuals with missing data on left ventricular ejection fraction, TyG-BMI, or USDD status. The overall prevalence of USDD in the study population was 33.6%, consistent with prior community-based estimates of subclinical diastolic dysfunction. The application of the 2025 ASE algorithmic diagnostic criteria (20) ensured that USDD classification reflected the most contemporary echocardiographic standards.

Participants were stratified into low- and high-TyG-BMI groups using the Youden index-derived optimal cutoff. As anticipated, participants in the high-TyG-BMI category exhibited significantly less favorable cardiometabolic profiles compared with their low-TyG-BMI counterparts. Specifically, the high-TyG-BMI group demonstrated elevated body mass index, higher systolic and diastolic blood pressure, increased triglyceride and fasting glucose concentrations, and reduced high-density lipoprotein cholesterol levels (Table 1). The prevalence of hypertension and diabetes mellitus was also significantly greater in the high-TyG-BMI stratum, consistent with the conceptualization of TyG-BMI as an integrative marker of metabolic dysfunction. Echocardiographic parameters, including left atrial volume index and the ratio of early transmitral flow velocity to early diastolic mitral annular velocity (E/e’), were significantly higher in the high-TyG-BMI group, while tissue Doppler-derived e${}^{\prime }$ velocities were correspondingly lower, indicating worse diastolic performance.

**Table 1:** Baseline characteristics of study participants by tyG-BMI group.

Characteristic	Overall (*N* = 1467)	Low TyG-BMI (< 220.000, *n* = 543)	High TyG-BMI (≥ 220.000, *n* = 924)	*P* value
Age (years)	59.00 (53.00, 67.00)	62.00 (54.00, 68.00)	59.00 (52.00, 67.00)	<0.001
Female, *n* (%)	601 (41.0%)	231 (42.5%)	370 (40.0%)	0.376
Current smoking, *n* (%)	320 (21.8%)	128 (23.6%)	192 (20.8%)	0.236
Alcohol use, *n* (%)	135 (9.2%)	36 (6.6%)	99 (10.7%)	0.012
BMI (kg/m²)	25.77 (23.57, 28.12)	22.99 (21.43, 24.07)	27.49 (25.95, 29.32)	<0.001
Waist circumference (cm)	88.00 (81.00, 95.00)	81.00 (76.00, 86.00)	93.00 (87.00, 98.00)	<0.001
SBP (mmHg)	151.00 (137.00, 162.50)	149.50 (132.75, 162.00)	152.00 (139.50, 162.50)	<0.001
DBP (mmHg)	90.00 (81.00, 100.50)	88.00 (79.50, 100.00)	91.50 (81.50, 101.00)	<0.001
TC (mmol/L)	5.21 (4.23, 6.43)	4.95 (4.06, 6.20)	5.39 (4.39, 6.51)	<0.001
LDL-C (mmol/L)	2.97 (2.20, 4.16)	2.79 (2.09, 3.90)	3.08 (2.29, 4.22)	0.001
HDL-C (mmol/L)	1.39 (1.20, 1.61)	1.47 (1.30, 1.73)	1.34 (1.16, 1.55)	<0.001
TG (mmol/L)	1.64 (1.15, 2.30)	1.20 (0.94, 1.55)	1.95 (1.45, 2.69)	<0.001
Glucose (mmol/L)	5.60 (5.30, 6.30)	5.40 (5.20, 5.90)	5.80 (5.30, 6.70)	<0.001
LVEF (%)	68.00 (63.00, 72.00)	68.00 (64.00, 72.00)	68.00 (63.00, 71.00)	0.188
LAVI (mL/m²)	27.40 (21.40, 33.88)	27.83 (21.37, 34.77)	27.07 (21.44, 33.50)	0.241
Average e’ (cm/s)	8.31 (6.86, 10.04)	8.86 (7.33, 10.44)	7.98 (6.64, 9.65)	<0.001
E/e’ ratio	8.12 (6.33, 10.17)	7.58 (6.08, 9.49)	8.48 (6.56, 10.51)	<0.001
Hypertension, *n* (%)	579 (39.5%)	168 (30.9%)	411 (44.5%)	<0.001
Diabetes, *n* (%)	128 (8.7%)	23 (4.2%)	105 (11.4%)	<0.001
CHD, *n* (%)	97 (6.6%)	39 (7.2%)	58 (6.3%)	0.572
Stroke, *n* (%)	165 (11.2%)	71 (13.1%)	94 (10.2%)	0.107
USDD, *n* (%)	493 (33.6%)	144 (26.5%)	349 (37.8%)	<0.001

Data are presented as median (25th, 75th percentile) or *n* (%). TyG-BMI cutoff=220.000 (Youden index).

BMI, body mass index; SBP, systolic blood pressure; DBP, diastolic blood pressure; TC, total cholesterol; LDL-C, low-density lipoprotein cholesterol; HDL-C, high-density lipoprotein cholesterol; TG, triglycerides; LVEF, left ventricular ejection fraction; LAVI, left atrial volume index; CHD, coronary heart disease; USDD, ultrasound-defined diastolic dysfunction.

### 3.2 Logistic regression analysis

Three sequentially adjusted logistic regression models were constructed to quantify the independent contribution of TyG-BMI to USDD risk (Table 2). In Model 1 (unadjusted), each 1-SD increment in TyG-BMI was significantly associated with elevated odds of USDD. This association persisted and was modestly strengthened in Model 2 after adjustment for age and sex, suggesting that demographic factors alone do not account for the observed relationship.

**Table 2:** Logistic regression analysis of tyG-BMI and USDD.

Model	OR	95% CI	*P* value	AUC
Continuous TyG-BMI (per 1-SD increase)				
Model 1 (Crude)	1.24	1.11–1.38	<0.001	—
Model 2 (Age/Sex)	1.37	1.22–1.53	<0.001	—
Model 3 (Fully adjusted)	1.35	0.42–4.40	0.616	—
Binary TyG-BMI (High vs. Low, cutoff=220.000)				
Model 1 (Crude)	1.68	1.33–2.12	<0.001	—
Model 2 (Age/Sex)	1.95	1.53–2.48	<0.001	—
Model 3 (Fully adjusted)	1.60	1.11–2.31	0.013	—

Model 1: Unadjusted. Model 2: Adjusted for age and sex. Model 3: Adjusted for age, sex, current smoking, alcohol use, hypertension, diabetes, CHD, stroke, BMI, TC, LDL-C, HDL-C, TG, and glucose. TyG-BMI cutoff=220.000 (Youden index).

OR, odds ratio; CI, confidence interval; AUC, area under the ROC curve.

In the fully adjusted Model 3, which incorporated age, sex, current smoking, alcohol consumption, hypertension, diabetes, coronary heart disease, stroke, body mass index, total cholesterol, low-density lipoprotein cholesterol, high-density lipoprotein cholesterol, triglycerides, and fasting glucose, the continuous association between TyG-BMI and USDD was partially attenuated. The binary comparison of high vs. low TyG-BMI, defined by the Youden-derived optimal threshold, demonstrated a significant independent association with USDD in the fully adjusted model. These findings indicate that TyG-BMI retains independent predictive information beyond its constituent metabolic components when analyzed as a categorical exposure, although the continuous association exhibited attenuation after comprehensive covariate adjustment.

### 3.3 ROC analysis and model discrimination

Receiver operating characteristic analysis was performed to assess the discriminative capacity of TyG-BMI for USDD across the three nested models (Figure 1). Model 1, incorporating TyG-BMI alone, yielded modest discrimination, consistent with the multifactorial etiology of diastolic dysfunction. The addition of age and sex in Model 2 produced a measurable improvement in the area under the ROC curve (AUC). The fully adjusted Model 3 achieved the highest AUC among the three specifications, indicating that TyG-BMI contributes incremental discriminatory information beyond conventional cardiovascular risk factors. Bootstrap resampling with 1,000 iterations was employed to generate 95% confidence intervals for each ROC curve, confirming the stability of the AUC estimates.

**Figure 1:**
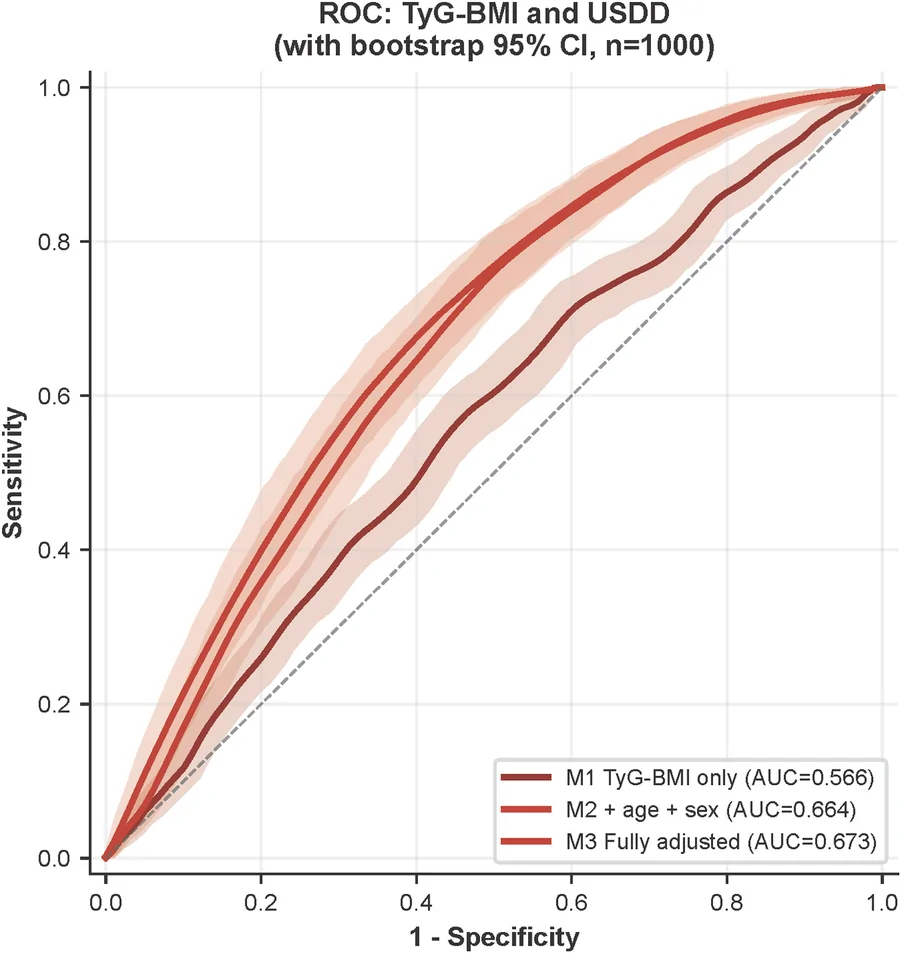
ROC curves for tyG-BMI predicting USDD across three models. Model 1: unadjusted TyG-BMI (AUC=0.566). Model 2: adjusted for age and sex (AUC=0.664). Model 3: fully adjusted model (AUC=0.673).

### 3.4 Dose-response analysis

Restricted cubic spline regression with knots positioned at the 10th, 50th, and 90th percentiles of the TyG-BMI distribution was performed to characterize the shape of the dose-response relationship (Figure 2). Across the total study population, a significant overall association was observed between TyG-BMI and the log-odds of USDD, without statistically significant evidence of departure from linearity. Subgroup-specific RCS analyses stratified by sex and age category revealed broadly consistent patterns, although the magnitude and precision of the estimated associations varied across strata. The spline analyses corroborated the logistic regression findings by demonstrating a monotonic increase in USDD risk across the TyG-BMI continuum. Although the per-1-SD estimate in the continuous model did not reach statistical significance, the monotonic increase observed in the spline analysis and the significantly higher odds in the high TyG-BMI group (≥ 220) together support a positive association, as further discussed in the Discussion section.

**Figure 2:**
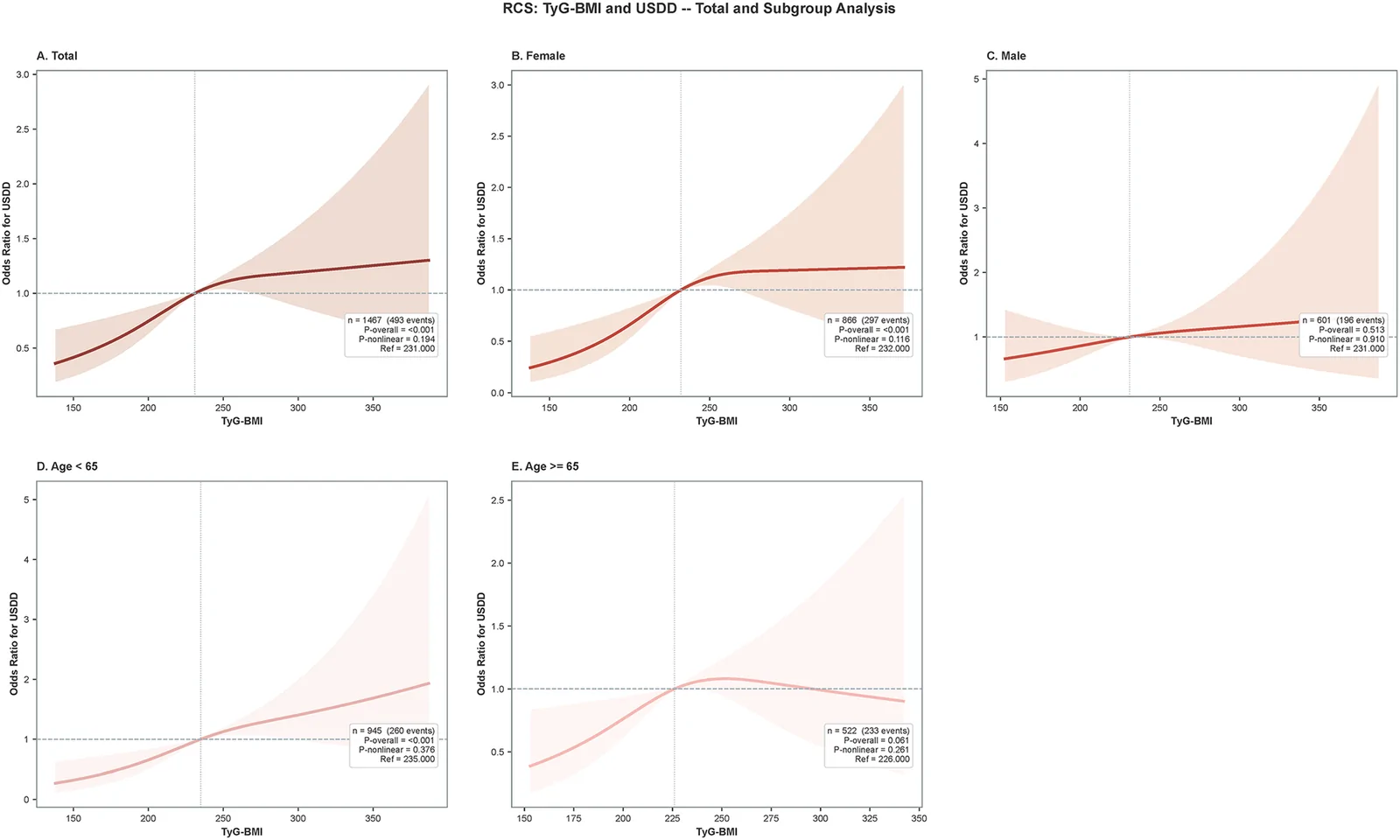
Restricted cubic spline analysis of the dose-response relationship between tyG-BMI and odds of USDD across total population and subgroups. Panel **A**: Total population (*n* = 1,467). Panel **B**: Female subgroup. Panel C: Male subgroup. Panel D: Age < 65 years. Panel E: Age ≥ 65 years. The solid curves represent the adjusted odds ratio, and the shaded areas represent 95% confidence intervals. Knots were placed at the 10th, 50th, and 90th percentiles of TyG-BMI.

### 3.5 LOESS regression of diastolic indices

Locally estimated scatterplot smoothing (LOESS) regression was employed to visualize the continuous associations between TyG-BMI and key echocardiographic parameters of diastolic function (Figure 3). Gradient-density scatter plots with superimposed LOESS curves and 95% bootstrap confidence bands revealed consistent patterns: higher TyG-BMI values were associated with progressively lower tissue Doppler e’ velocities, reflecting impaired myocardial relaxation, and progressively higher E/e’ ratios, indicating elevated left ventricular filling pressure. These gradients were evident across all four diastolic indices examined, providing convergent evidence that TyG-BMI tracks with the severity of subclinical diastolic impairment along a continuous spectrum.

**Figure 3:**
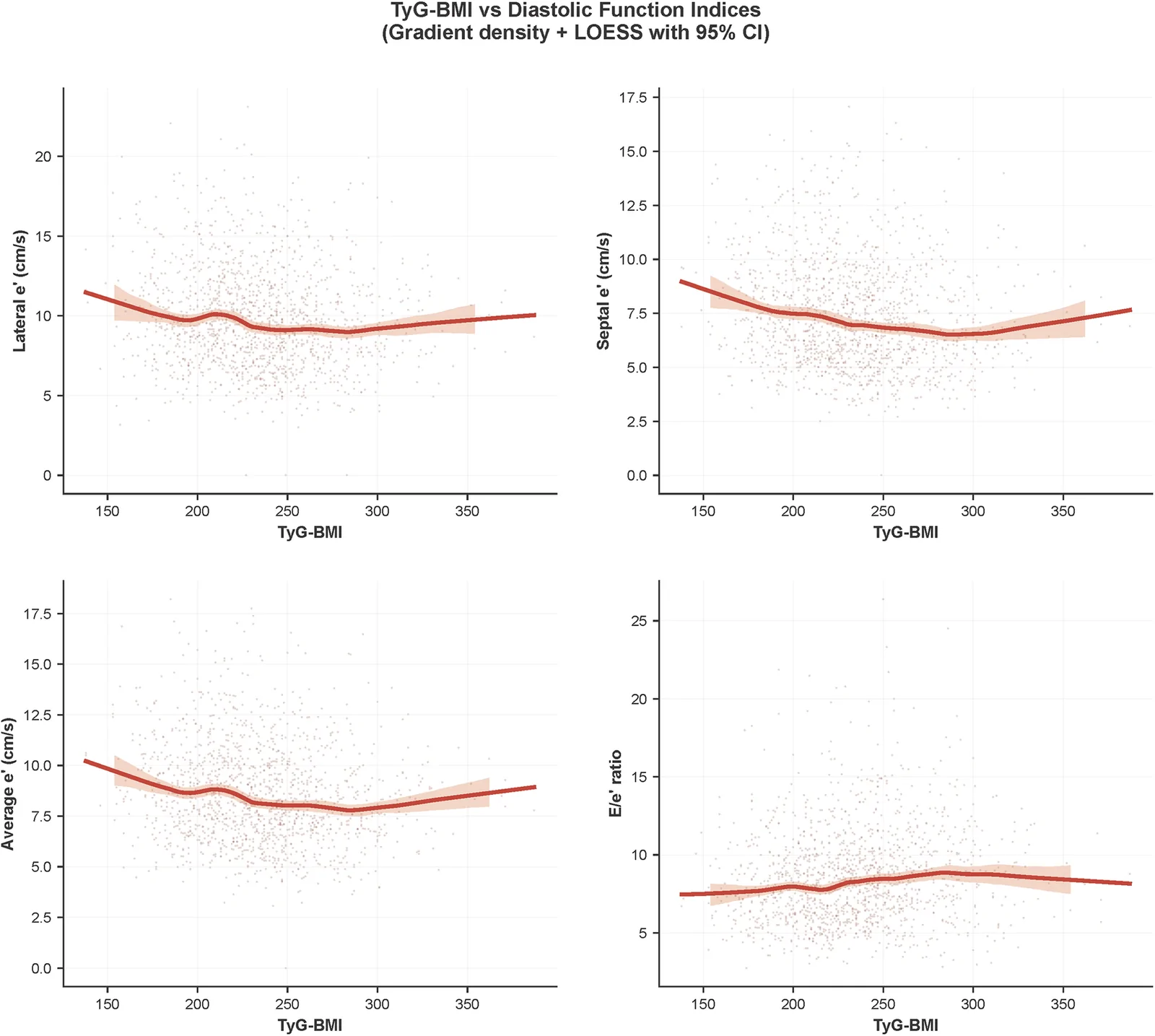
LOESS regression plots showing the association between tyG-BMI and continuous diastolic parameters: lateral e′ velocity, septal e′ velocity, average e′ velocity, and the E/e′ ratio.

### 3.6 Subgroup and interaction analyses

Prespecified subgroup analyses were conducted to evaluate the consistency of the TyG-BMI–USDD association across clinically relevant population strata (Figure 4). Forest plots for both continuous (per 1-SD) and binary (high vs. low) exposures demonstrated broadly consistent directionality of effect across subgroups defined by age, sex, hypertension status, diabetes status, smoking status, alcohol consumption, coronary heart disease, and stroke history. Interaction terms between TyG-BMI and each stratification variable were tested in the fully adjusted model. No statistically significant interactions were detected for age (P for interaction=0.390), sex (0.731), hypertension (0.478), current smoking (0.784), alcohol use (0.181), CHD (0.392), or stroke (0.086); the interaction with diabetes was of marginal significance (*P* = 0.048). Quartile-based interaction analyses with continuous clinical covariates (SBP: 0.895; DBP: 0.261; TC: 0.094; LDL-C: 0.016; TG: 0.093; HDL-C: 0.357) further confirmed the absence of substantial effect modification, with only the LDL-C interaction reaching nominal significance (Figure 5), suggesting that the association between TyG-BMI and USDD is relatively homogeneous across these demographic and clinical categories.

**Figure 4:**
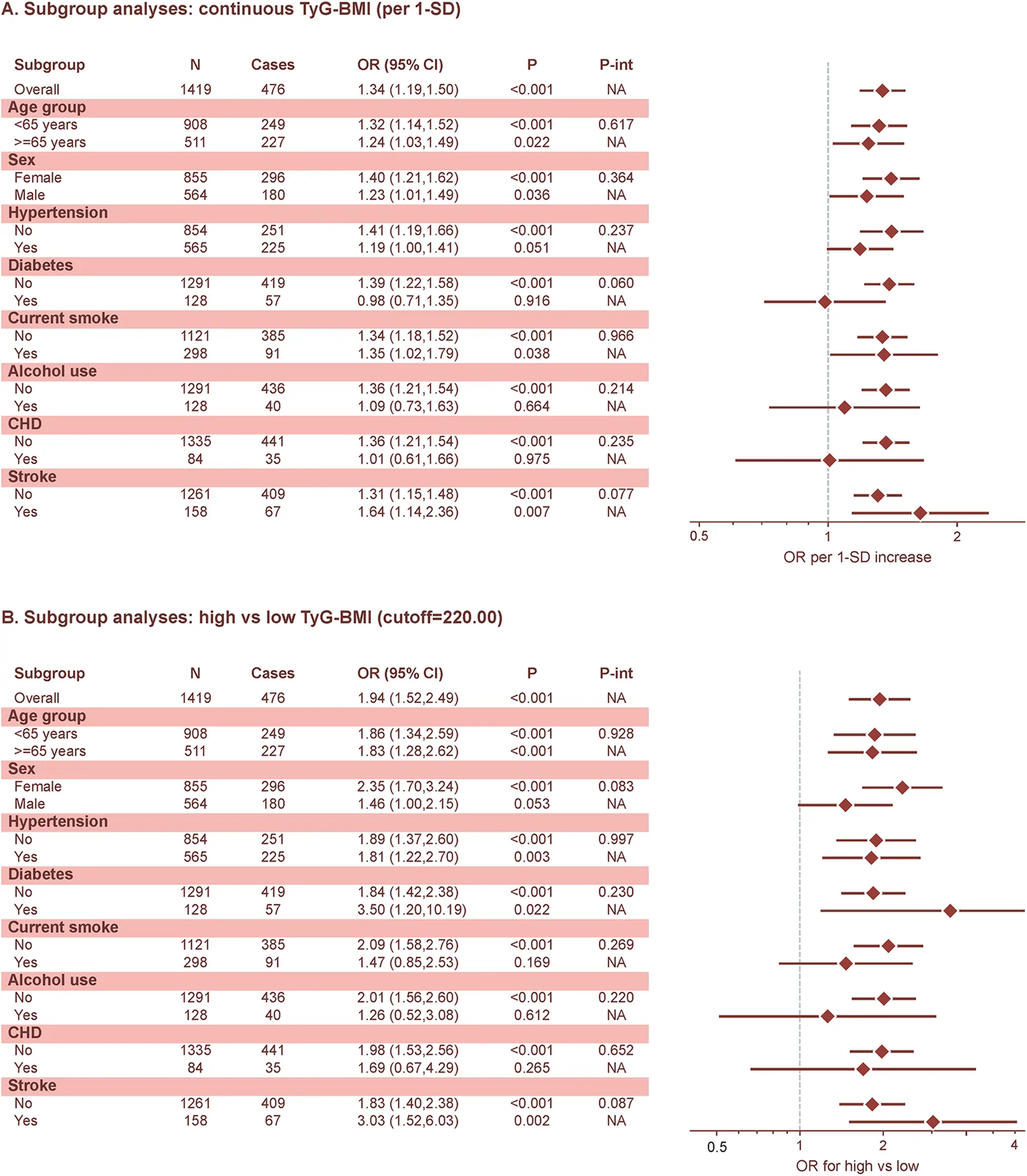
Subgroup analyses of the association between tyG-BMI and USDD. **(A)** Forest plot for continuous TyG-BMI (per 1-SD increase); **(B)** forest plot for high vs. low TyG-BMI (cutoff=220.000). Estimates are adjusted for age and sex within each stratum, where applicable. *P* values for interaction are shown; no statistically significant interactions were detected except for diabetes (*P* = 0.048) and LDL-C (*P* = 0.016).

**Figure 5:**
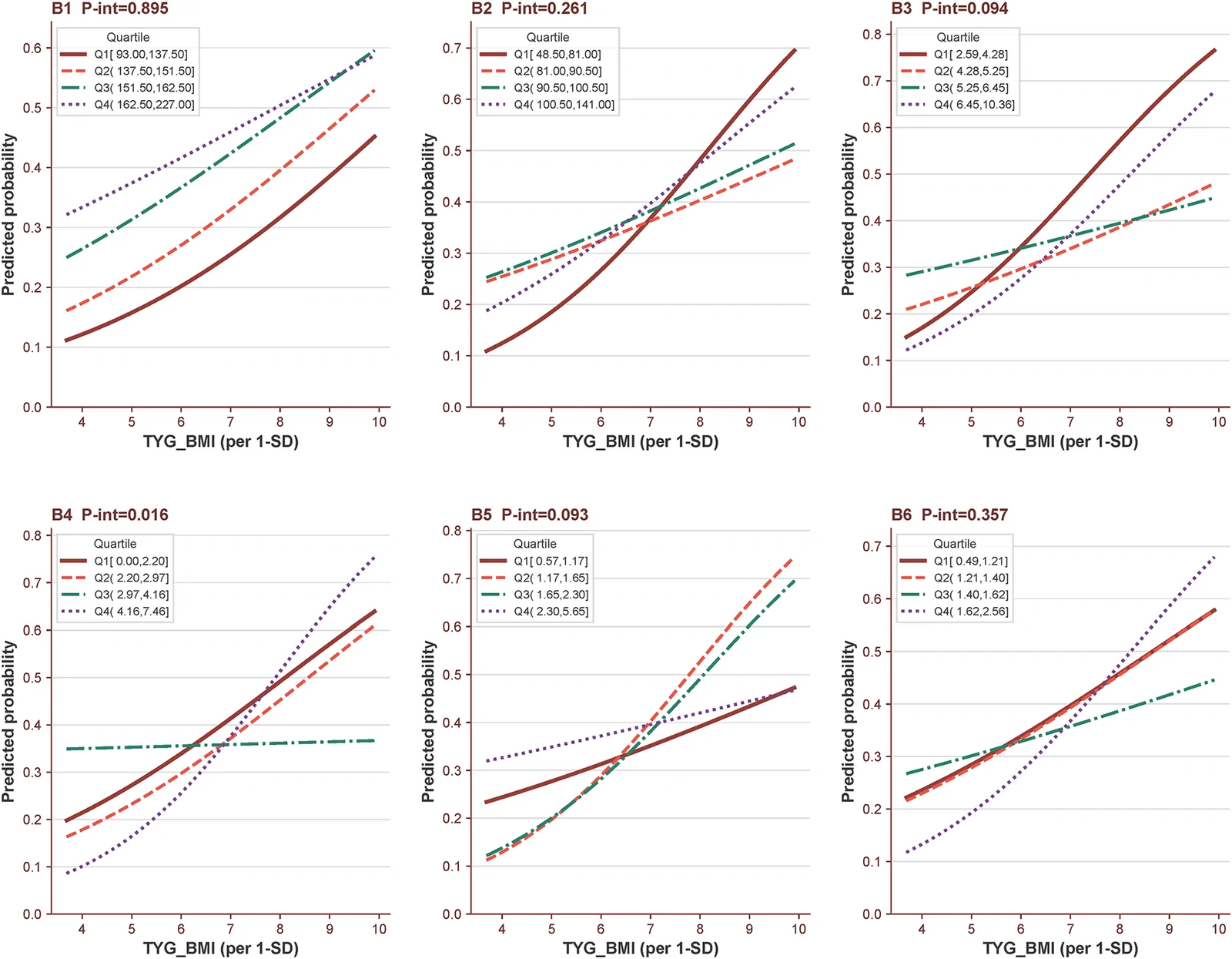
Interaction analyses of tyG-BMI with continuous clinical covariates for USDD. Predicted probabilities of USDD across quartiles of (**B1**) SBP, (**B2**) DBP, (**B3**) TC, (**B4**) LDL-C, (**B5**) TG, and (**B6**) HDL-C, with *P* values for interaction shown in each panel.

### 3.7 Machine learning validation

To corroborate the regression-based findings with an independent computational framework, nine supervised machine learning classifiers were benchmarked under 5-fold stratified cross-validation (Figure 6). LASSO regression with cross-validated regularization parameter selection identified a parsimonious set of predictive features for USDD, among which TyG-BMI was retained, confirming its independent predictive contribution. Least absolute shrinkage and selection operator (LASSO) regression with cross-validated regularization parameter selection identified a parsimonious set of seven predictive features for USDD—age, systolic blood pressure, waist circumference, HDL-C, coronary heart disease, stroke, and TyG-BMI—among which TyG-BMI was retained, confirming its independent predictive contribution, whereas sex, DBP, BMI, TC, TG, LDL-C, glucose, smoking, alcohol use, hypertension, and diabetes were excluded. Variance inflation factor diagnostics showed that, apart from the expected high collinearity between BMI and TyG-BMI (which arises because BMI is a component of TyG-BMI), the remaining candidate predictors had VIF values within acceptable limits. Among the classifiers evaluated, tree-based ensemble methods showed the best discriminative performance; nevertheless, the overall discrimination was moderate, with cross-validated AUC values ranging from 0.59 to 0.67 (Figure 6D), consistent with the logistic regression models.

**Figure 6:**
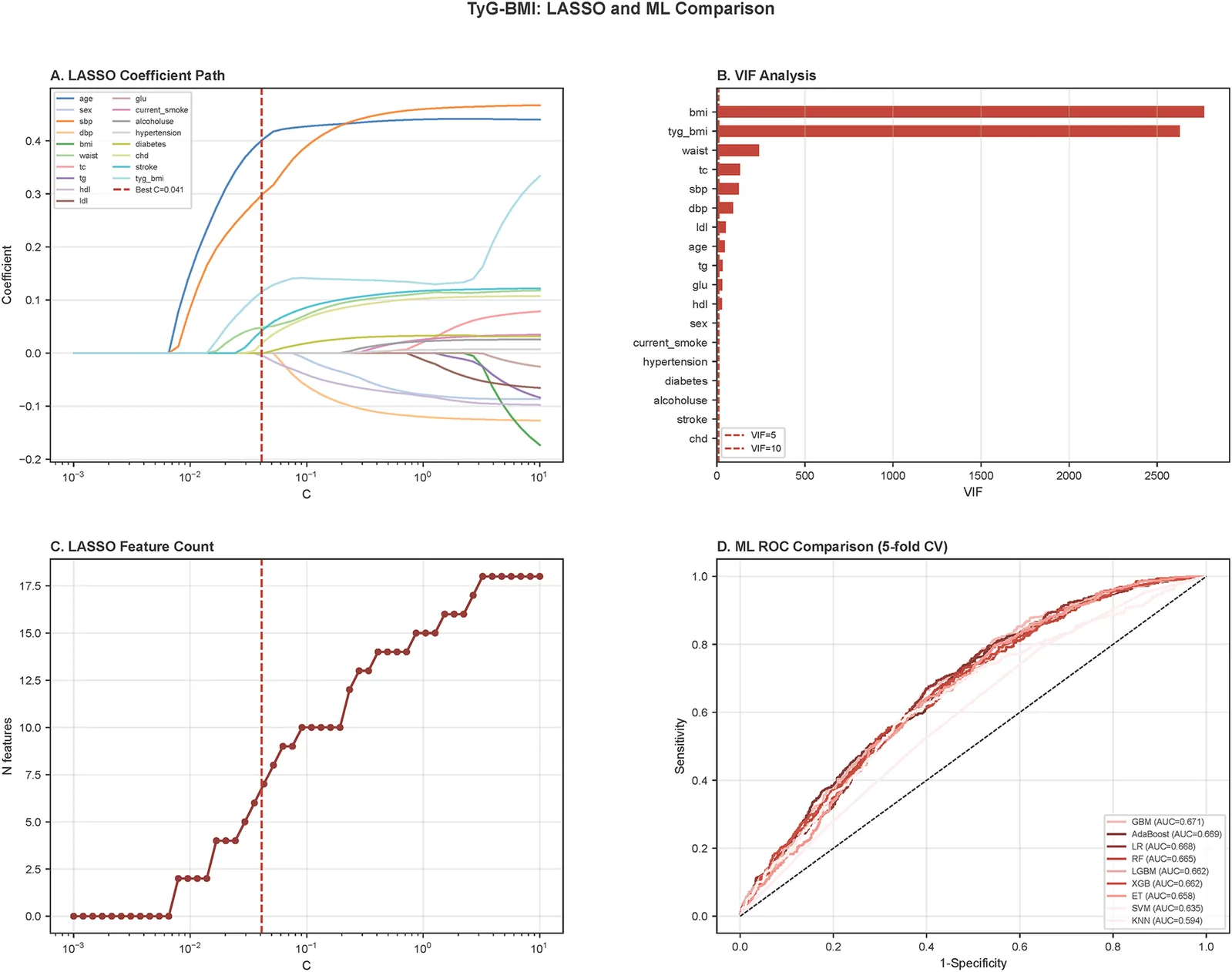
LASSO feature selection, variance inflation factor (VIF) analysis, and machine learning model comparison. **(A)** LASSO coefficient paths for the candidate predictors; the optimal penalty parameter C was selected by 5-fold cross-validation using the ROC AUC. **(B)** VIF analysis; apart from the expected high collinearity between BMI and TyG-BMI (which arises because BMI is a component of TyG-BMI), the remaining candidate predictors had VIF values within acceptable limits. **(C)** Number of features selected across regularization levels. **(D)** Cross-validated ROC curves of the nine machine learning classifiers (AUC 0.59–0.67).

### 3.8 SHAP interpretability analysis

SHapley Additive exPlanations (SHAP) analysis was performed on the best-performing XGBoost model to quantify per-feature contributions to USDD prediction (Figure 7). The SHAP rose chart, beeswarm summary plot, and bar importance ranking showed that age, systolic blood pressure, and BMI contributed most to model predictions, with TyG-BMI ranking fourth among the top five predictors, indicating a moderate but non-dominant contribution to USDD prediction. The SHAP dependence plots for TyG-BMI exhibited a broadly positive relationship with predicted USDD probability, corroborating the directionality observed in the logistic regression and spline analyses. Individual-level SHAP values revealed heterogeneity in the magnitude of TyG-BMI’s contribution across participants, suggesting that the predictive value of TyG-BMI may vary according to the background cardiometabolic context.

**Figure 7:**
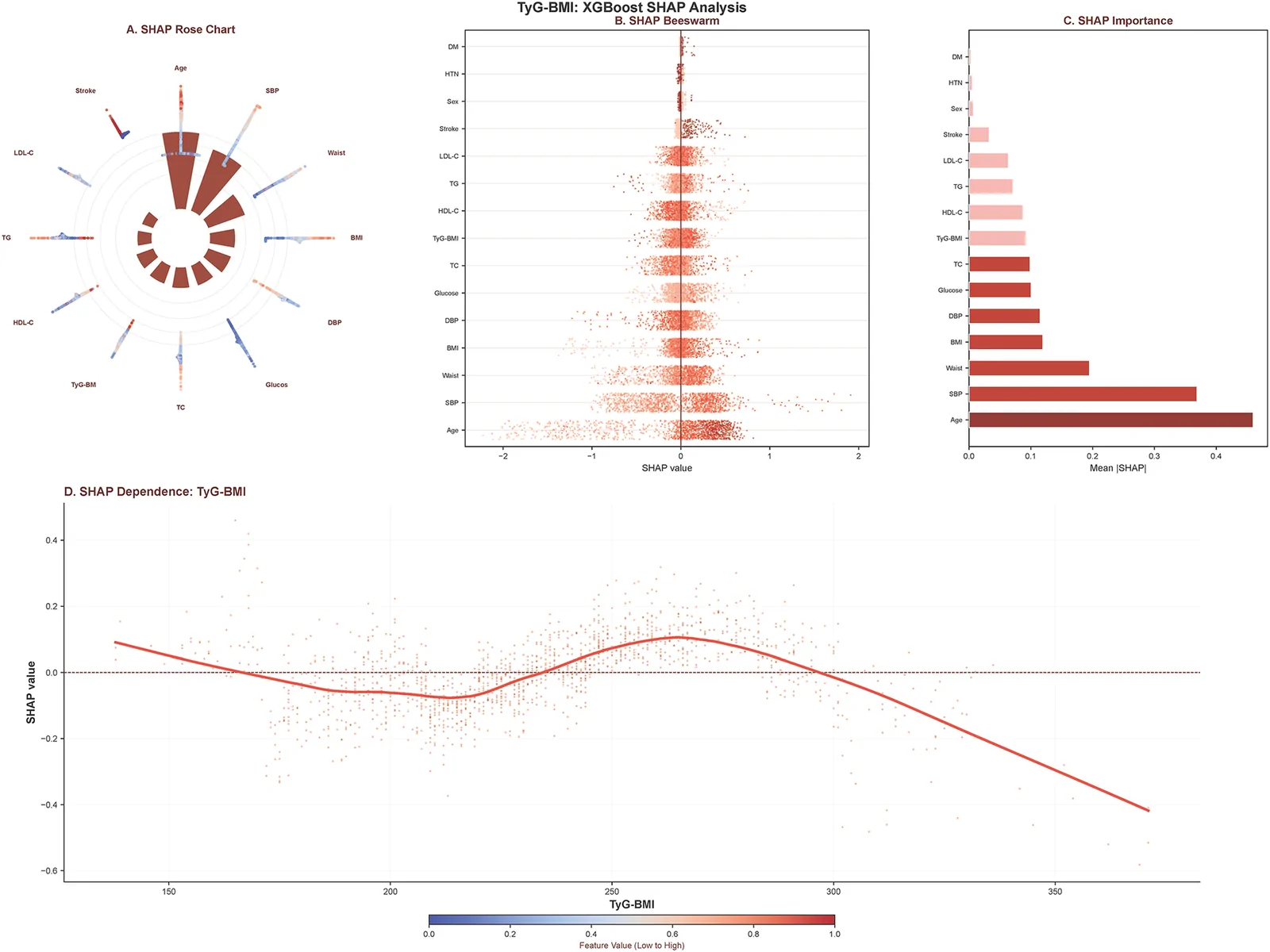
SHAP analysis from the XGBoost model. **(A)** SHAP rose chart showing the feature importance ranking. The top five predictors were age, SBP, waist circumference, BMI, and DBP. **(B)** SHAP beeswarm plot. **(C)** Mean SHAP importance ranking. **(D)** SHAP dependence plot for TyG-BMI.

## 4 Discussion

### 4.1 Principal findings

This cross-sectional investigation of community-dwelling Chinese adults who underwent comprehensive echocardiographic phenotyping and metabolic profiling provides several lines of evidence regarding the relationship between TyG-BMI and ultrasound-defined diastolic dysfunction. First, TyG-BMI demonstrated a graded, positive association with USDD in unadjusted and demographic-adjusted logistic regression models, with the binary high-vs.-low comparison retaining statistical significance after comprehensive multivariable adjustment. Second, restricted cubic spline analysis confirmed a significant overall dose-response relationship across the TyG-BMI continuum, with broadly consistent patterns observed across sex- and age-stratified subgroups. It should be noted that the results of the continuous and binary analyses of TyG-BMI seem inconsistent at first glance. In the fully adjusted model, TyG-BMI as a continuous variable was not significantly associated with USDD (per 1-SD: OR=1.35, 95% CI 0.42–4.40, *P* = 0.616), whereas high TyG-BMI (≥ 220) was significantly associated with an increased risk (OR=1.60, 95% CI 1.11–2.31, *P* = 0.013). The continuous estimate was in the expected positive direction but had a wide confidence interval, indicating limited statistical precision. In addition, continuous variables are more sensitive to measurement noise and distributional skewness than a binary split, which provides a more stable between-group contrast. Notably, the continuous association was statistically significant in the crude (OR=1.24, 95% CI 1.11–1.38, *P* < 0.001) and demographic-adjusted (OR=1.37, 95% CI 1.22–1.53, *P* < 0.001) models but became weaker in the fully adjusted model (Model 3: age, sex, current smoking, alcohol use, hypertension, diabetes, CHD, stroke, BMI, TC, LDL-C, HDL-C, TG, and glucose), suggesting that these closely related metabolic factors and comorbidities partly explain the weaker association on the continuous scale. Importantly, the restricted cubic spline analysis showed a significant overall association (P for overall < 0.001) without a significant departure from linearity (P for non-linearity=0.194), and the machine-learning analyses also confirmed the predictive contribution of TyG-BMI, indicating that the association is positive and essentially monotonic rather than threshold-dependent or absent. From a clinical perspective, the ROC-derived threshold of 220 may serve as a practical reference for identifying middle-aged and elderly individuals at higher risk of left ventricular diastolic dysfunction, although its generalizability needs further validation. Third, LOESS regression revealed consistent gradients between TyG-BMI and continuous echocardiographic indices of diastolic function, including tissue Doppler velocities, filling pressure estimates, and left atrial volume. Fourth, machine learning analyses employing LASSO-based feature selection and XGBoost-SHAP interpretability identified TyG-BMI as one of the top-five predictors of USDD, although its contribution was smaller than those of age, systolic blood pressure, and BMI, corroborating a moderate—rather than dominant—role in USDD prediction through complementary computational approaches. Collectively, these convergent results support the utility of TyG-BMI as an integrative biomarker for subclinical diastolic dysfunction.

### 4.2 Comparison with previous studies

The present findings align with and extend the existing literature on metabolic indices and cardiovascular risk stratification. Previous investigations have established that insulin resistance markers, including the triglyceride-glucose index and its anthropometry-adjusted derivatives, are associated with adverse cardiovascular outcomes including incident coronary artery disease, stroke, and cardiovascular mortality across diverse populations (22, 23). These findings are supported by prospective cohort evidence from Chinese populations: in a nationwide cohort of 3.5 million Chinese adults (ChinaHEART), an elevated TyG index above the cut-off was associated with markedly increased all-cause and cardiovascular mortality (24), and in the CHARLS cohort of middle-aged and older adults, a higher cumulative average TyG-BMI was significantly associated with incident cardiovascular disease (25). However, the specific relationship with echocardiographically-defined diastolic dysfunction—a critical precursor to heart failure with preserved ejection fraction—has received comparatively less systematic attention in biomarker research.

The observed association between TyG-BMI and USDD is consistent with mechanistic studies demonstrating that insulin resistance promotes adverse myocardial remodeling through interconnected pathways involving activation of the renin-angiotensin-aldosterone system, increased oxidative stress, advanced glycation end-product formation, and pro-inflammatory cytokine signaling (4, 26–28). Composite indices such as TyG-BMI may offer superior risk discrimination compared with single-component markers because they simultaneously capture multiple dimensions of metabolic dysfunction, thereby better reflecting the systemic and multifactorial nature of cardiometabolic disease (29, 30).

The modest AUC values observed in the ROC analyses are consistent with the multifactorial etiology of diastolic dysfunction and are comparable to those reported for other metabolic biomarkers in community-based studies (6, 7). It is important to note that TyG-BMI is not proposed as a standalone diagnostic tool but rather as a screening adjunct that may complement existing risk assessment strategies.

### 4.3 Potential mechanisms

Several interconnected pathophysiological pathways may mechanistically link TyG-BMI to subclinical diastolic dysfunction. First, insulin resistance at the cardiomyocyte level impairs myocardial glucose uptake through downregulation of glucose transporter type 4 (GLUT4) translocation, shifting substrate utilization toward fatty acid oxidation and promoting intracellular lipid accumulation, or cardiac steatosis (31, 32). Excessive myocardial lipid intermediates, including diacylglycerols and ceramides, can activate protein kinase C isoforms and promote mitochondrial dysfunction, contributing to impaired cardiomyocyte relaxation and reduced ventricular compliance.

Second, the chronic low-grade systemic inflammation that characterizes insulin-resistant states contributes to coronary microvascular endothelial dysfunction through reduced nitric oxide bioavailability, increased expression of vascular adhesion molecules, and enhanced leukocyte-endothelial interactions (4, 5). Coronary microvascular inflammation is now recognized as a central mechanism in the pathogenesis of HFpEF, whereby endothelial activation in the myocardial microvasculature triggers downstream fibrotic signaling cascades in adjacent cardiomyocytes and interstitial fibroblasts.

Third, the pro-fibrotic milieu associated with metabolic dysfunction, characterized by elevated transforming growth factor-beta (TGF-β), connective tissue growth factor (CTGF), and galectin-3, promotes excessive deposition of extracellular matrix proteins, including collagen types I and III, within the myocardial interstitium (33, 34). Increased myocardial collagen content and collagen cross-linking directly increase passive myocardial stiffness, impairing diastolic relaxation and elevating left ventricular filling pressures. Additionally, titin hypophosphorylation—a post-translational modification associated with insulin resistance—increases the passive tension of cardiomyocytes, further contributing to diastolic dysfunction at the sarcomere level.

The superior performance of composite indices such as TyG-BMI may reflect their ability to simultaneously quantify adiposity-related and lipid-related dimensions of metabolic stress. Adipose tissue expansion, particularly visceral and epicardial adiposity, contributes to diastolic dysfunction through pericardial fat deposition, paracrine secretion of pro-inflammatory adipokines, and hemodynamic overload from expanded intravascular volume (35, 36). Concurrently, atherogenic dyslipidemia—characterized by elevated triglycerides, small dense low-density lipoprotein particles, and reduced high-density lipoprotein cholesterol—promotes systemic endothelial dysfunction and microvascular rarefaction, further compromising myocardial perfusion and function (37, 38).

### 4.4 Clinical implications

These findings carry several potential implications for clinical practice and public health. First, TyG-BMI may serve as a practical, low-cost screening index for identifying community-dwelling individuals at elevated risk of subclinical diastolic dysfunction, particularly in resource-limited settings where comprehensive echocardiography may not be readily accessible (39). The routine availability of the component measurements required for TyG-BMI calculation—typically part of standard metabolic panels and anthropometric assessments—enhances its feasibility for large-scale screening applications. However, the TyG-BMI threshold of 220 was derived from a single-center community cohort and has not been externally validated; therefore, this threshold and the proposed screening application should be regarded as exploratory and require confirmation in independent, multi-center, and diverse populations before any clinical or community-wide use can be recommended.

Second, the identification of modifiable metabolic risk factors through composite indices such as TyG-BMI could inform targeted preventive strategies. This is particularly relevant given the high prevalence of dyslipidemia and related metabolic abnormalities in at-risk populations, such as patients with hypertension (40). Lifestyle interventions including dietary modification, increased physical activity, and weight reduction have been shown to improve both insulin sensitivity and echocardiographic measures of diastolic function (41, 42). Among dietary strategies, polyphenol-rich diets have been shown to favorably modulate lipid metabolism and may further improve the metabolic profile (43). More broadly, lifestyle and environmental factors are recognized as key determinants of cardiovascular health (44). Pharmacological interventions targeting insulin resistance, including metformin and sodium-glucose cotransporter-2 inhibitors, represent additional therapeutic avenues that warrant investigation in the context of diastolic dysfunction prevention (45).

Third, the integration of TyG-BMI into existing cardiovascular risk prediction algorithms may enhance stratification beyond traditional risk factors, potentially enabling earlier identification of individuals who would benefit from intensified preventive cardiology interventions. However, the incremental predictive value of TyG-BMI over established risk scores requires formal evaluation in prospective studies before clinical implementation can be recommended.

### 4.5 Study strengths

This investigation has several notable strengths that enhance confidence in the reported findings. The community-based sampling frame, as opposed to hospital-based or referral populations, enhances the generalizability of the results to the broader population of middle-aged and older Chinese adults. Diastolic function was rigorously phenotyped using the contemporary 2025 American Society of Echocardiography algorithmic approach (20), representing the most current diagnostic standard and ensuring alignment with clinical practice guidelines. The analytic strategy employed triangulation across multiple complementary statistical frameworks—conventional logistic regression, flexible spline-based dose-response modeling, locally estimated scatterplot smoothing, stratified subgroup analyses with formal interaction testing, and machine learning approaches, performed as complementary and exploratory analyses, provided convergent evidence consistent with the primary regression and spline analyses. The application of SHapley Additive exPlanations interpretability analysis offers granular, per-feature insights into model predictions, extending beyond traditional regression outputs and enabling visualization of the directionality and magnitude of individual predictor contributions (46). All echocardiographic measurements were performed by credentialed sonographers following standardized protocols with blinded interpretation, minimizing the potential for measurement bias.

### 4.6 Study limitations

Several limitations warrant careful consideration when interpreting these results. First, the cross-sectional design fundamentally precludes causal inference regarding the temporal relationship between TyG-BMI elevation and the development of diastolic dysfunction. While the observed associations are biologically plausible and supported by mechanistic evidence, prospective longitudinal studies with serial measurements of both TyG-BMI and diastolic function are required to establish temporality.

Second, the study population was recruited from a single geographic region in central China (Luohe, Henan Province). Although this enhances internal validity through standardized recruitment and measurement protocols, it may limit the generalizability of the findings to populations with different genetic backgrounds, dietary patterns, lifestyle factors, or cardiometabolic risk profiles. Multi-ethnic, multi-region replication studies are warranted. Furthermore, the TyG-BMI threshold of 220 was derived from a single-center community cohort and has not been externally validated; therefore, the generalizability of this threshold to other populations remains to be established. Therefore, this threshold should not be directly generalized to other ethnic or geographic populations or treated as a formal clinical diagnostic threshold until it is confirmed in independent cohorts.

Third, while transthoracic echocardiography represents the accepted clinical standard for the non-invasive assessment of diastolic function, invasive hemodynamic measurements using conductance catheters remain the gold standard for quantifying left ventricular filling pressures and chamber stiffness (47). The potential for misclassification of diastolic function, although minimized through the application of standardized 2025 ASE criteria, cannot be entirely excluded, as invasive hemodynamic measurements were not available in this community-based cohort.

Fourth, despite comprehensive multivariable adjustment for a broad panel of demographic, clinical, and biochemical covariates, the possibility of residual confounding from unmeasured or incompletely measured variables—including dietary quality, physical activity patterns, socioeconomic factors, and genetic predisposition—cannot be excluded.

Fifth, the modest AUC values observed for TyG-BMI-based models across all three specifications indicate that TyG-BMI alone is insufficient for definitive clinical diagnosis of diastolic dysfunction. TyG-BMI should be conceptualized as a screening adjunct or risk enrichment tool rather than as a diagnostic test, and its clinical utility should be evaluated as part of a broader, multi-marker risk assessment strategy. Finally, the machine-learning analyses are purely associative and cannot establish causality, consistent with the cross-sectional nature of the dataset.

### 4.7 Future directions

Several avenues for future research emerge from these findings. Prospective cohort studies with longitudinal follow-up and serial echocardiographic assessments are needed to determine whether TyG-BMI independently predicts incident diastolic dysfunction and, critically, progression from subclinical diastolic abnormalities to overt heart failure with preserved ejection fraction. Intervention studies—whether randomized controlled trials of lifestyle modification or pharmacological therapies targeting insulin resistance—that examine whether improvements in TyG-BMI translate to measurable and clinically meaningful improvements in diastolic function would substantially strengthen causal inference.

Multi-ethnic validation studies conducted in geographically and demographically diverse populations are essential to assess the consistency, transportability, and potential heterogeneity of the TyG-BMI–USDD association across different epidemiological contexts. Investigations examining the integration of TyG-BMI with emerging circulating biomarkers of cardiac stress and injury, including high-sensitivity cardiac troponins, natriuretic peptides, and novel inflammatory and fibrotic markers, may reveal whether multi-marker panels yield incremental predictive value for subclinical cardiac dysfunction beyond that provided by TyG-BMI alone (48, 49). Finally, cost-effectiveness analyses evaluating TyG-BMI-based screening strategies against current standard-of-care approaches would inform decisions regarding potential clinical implementation.

## Data Availability

The raw data supporting the conclusions of this article will be made available by the authors, without undue reservation.
